# A review of the pharmaceutical exposome in aquatic fauna^☆^

**DOI:** 10.1016/j.envpol.2018.04.012

**Published:** 2018-08

**Authors:** Thomas H. Miller, Nicolas R. Bury, Stewart F. Owen, James I. MacRae, Leon P. Barron

**Affiliations:** a Analytical & Environmental Sciences Division, Faculty of Life Sciences and Medicine, King's College London, 150 Stamford Street, London, SE1 9NH, United Kingdom; b Faculty of Science, Health and Technology, University of Suffolk, James Hehir Building, University Avenue, Ipswich, Suffolk, IP3 0FS, UK; c Division of Diabetes and Nutritional Sciences, Faculty of Life Sciences and Medicine, King's College London, Franklin Wilkins Building, 150 Stamford Street, London, SE1 9NH, UK; d AstraZeneca, Global Environment, Alderley Park, Macclesfield, Cheshire SK10 4TF, UK; e Metabolomics Laboratory, The Francis Crick Institute, 1 Midland Road, London, NW1 1AT, UK

**Keywords:** Occurrence, Pharmaceuticals, Fish, Bioconcentration, Invertebrates

## Abstract

Pharmaceuticals have been considered ‘contaminants of emerging concern’ for more than 20 years. In that time, many laboratory studies have sought to identify hazard and assess risk in the aquatic environment, whilst field studies have searched for targeted candidates and occurrence trends using advanced analytical techniques. However, a lack of a systematic approach to the detection and quantification of pharmaceuticals has provided a fragmented literature of serendipitous approaches. Evaluation of the extent of the risk for the plethora of human and veterinary pharmaceuticals available requires the reliable measurement of trace levels of contaminants across different environmental compartments (water, sediment, biota - of which biota has been largely neglected). The focus on pharmaceutical concentrations in surface waters and other exposure media have therefore limited both the characterisation of the exposome in aquatic wildlife and the understanding of cause and effect relationships. Here, we compile the current analytical approaches and available occurrence and accumulation data in biota to review the current state of research in the field. Our analysis provides evidence in support of the ‘Matthew Effect’ and raises critical questions about the use of targeted analyte lists for biomonitoring. We provide six recommendations to stimulate and improve future research avenues.

## Highlights

• Occurrence and accumulation of pharmaceuticals in aquatic fauna is presented.
• Evidence of the Matthew Effect from biased pharmaceutical pre-selection.
• Field versus laboratory derived accumulation factors showed a large disparity.
• Specific accumulation is pronounced for liver and bile over other tissues.
• Recommendations for advancements within environmental toxicology are proposed.

## Graphical abstract

**Figure:**
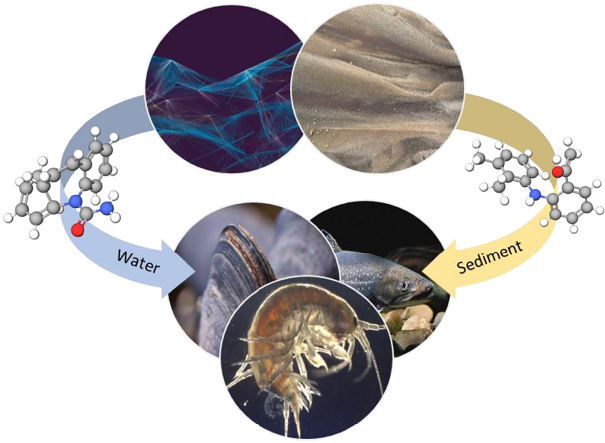

## Teaser

The determination of the pharmaceutical exposome in aquatic fauna is critical to understanding the extent of contamination and the potential risk in the environment.

## 1 Pharmaceuticals as a cause for concern in the aquatic environment

Chemical contaminants entering the environment are a consistent cause for concern. In particular, pharmaceutical and personal care products (PPCPs) have been identified as emerging contaminants (Daughton and Ternes, 1999; Ellis, 2006), i.e. compounds which are not routinely monitored and are suspected to cause adverse effects in the environment. In 2015, at the International Conference on Chemicals Management (ICCM), the pharmaceutical industry and non-governmental bodies agreed that the environment now requires protection from “pharmaceutical pollution” (Time to get clean. Nature, 2015). The combination of total compounds exposed to, and their effects on, an organism over an entire life cycle is termed the ‘exposome’ (Rappaport, 2011; Escher and Hermens, 2004). Global occurrence and fate in abiotic aqueous (Balakrishna et al., 2017; Bu et al., 2013; Heberer, 2002) and solid matrices (Díaz-Cruz et al., 2003; Halling-Sørensen et al., 1998; Pan et al., 2009; Tadeo et al., 2012) have formed the focus of several in-depth reviews. In the context of the exposome - especially pharmaceutical residues - it is arguably the internalised compound concentrations that will determine biological effects in an organism. To date, biomonitoring of pharmaceuticals in aquatic biota (as potentially the most at risk group) have not been reviewed in great depth as studies have only relatively recently begun to emerge more frequently in the literature. However, we direct the reader to earlier literature from 2011, that covers a wide range of contaminants in aquatic wildlife including the very first studies associated with the measurement of pharmaceuticals (Beyer and Meador, 2011).

The EU Water Framework Directive has included pharmaceuticals on a dynamic ‘watch-list’ based on potential for adverse effects in the aquatic environment. This list includes insecticides, herbicides, a sunscreen, several antibiotics, some natural hormones and two pharmaceuticals (17-alpha-Ethinylestradiol (EE2) from the birth control pill and diclofenac, a non-steroidal inflammatory drug) under the Environmental Quality Standards Directive and are subject to European monitoring (Carvalhoet al, 2015). The Convention for the Protection of the Marine Environment of the North-East Atlantic (OSPAR) was the first body to formally recognise pharmaceutical contamination, where the compound clotrimazole was included on their priority action list (OSPAR, 2002). OSPAR now lists 28 substances or groups of substances, with a further 264 compounds (including 25 pharmaceuticals) as contaminants of possible concern under four separate categories (OSPAR). Several non-regulatory groups, such as the Network of reference laboratories, research centres and related organisations for monitoring of emerging environmental substances (NORMAN), share knowledge on environmental contaminants gathered from monitoring campaigns and aim to harmonise analytical approaches for contaminant identification and determination in a range of environmental compartments (Brack et al., 2012). Other groups include those such as the United Nations Educational, Scientific, and Cultural Organization (UNESCO) which have recently released a series on ‘emerging pollutants in water’ (UNESCO and HELCOM, 2017). Whilst, the collation and dissemination of information from these groups is valuable, it is also essential that reported information is consistent and accurate otherwise it can lead to reduced data value. Therefore, it may be prudent to standardise units when reporting occurrence data (e.g. μg L^−1^) to avoid this type of error. As observed in other fields, reporting to accepted quality standards in research articles is critical if they are used to help inform policy, scientific practice and knowledge (Munafò et al., 2017). However, research articles can omit critical information relevant to the study and this potentially decrease their value. Appropriate chemical analysis method validation guidelines should be used, ideally integrated within the wider umbrella of acceptable reporting guidelines, and this would help ensure the reliability of any reported contaminant concentrations in biota. A range of guidelines are available that have been developed to improve reporting standards across health research (Simera et al., 2010) and this should ideally be no different for ecotoxicology. For example, guidelines such as Animals in Research: Reporting In Vivo Experiments (ARRIVE) (Kilkenny et al., 2010) could be adapted to improve the reporting standards for monitoring campaigns and effect-based studies (especially those using bespoke behavioural endpoints). However, guidelines for both method validation and reporting standards still require consensus within the scientific community in this particular field.

Pharmaceutical concentrations in environmental waters are generally considered non-toxic to humans directly (ng-μg L^−1^), but this may not be the case for wildlife. Unlike other traditional persistent organic pollutants, PPCPs are not so easily classified as they are not always persistent. They are, however, pseudo-persistent due to continual influx to the environment from several sources, including waste water treatment plants (WWTPs), manufacturing, agriculture and aquaculture, amongst other routes (Boxall et al., 2012). Furthermore, pharmaceuticals are generally designed not to be bioaccumulative (Lipinski et al., 1997), as demonstrated during *in vivo* laboratory exposures (Miller et al, 2016, 2017; Meredith-Williams et al., 2012; Nichols et al., 2015; Nallani et al., 2011). Effects are often studied and observed at non-environmentally relevant concentrations of single compounds (i.e. acute toxicity) under defined laboratory conditions (Carlsson et al., 2006). Any effects observed are generally not explicitly linked to the cause (i.e. the internalised drug is not determined) (Rand-Weaver et al., 2013). Pharmaceutical residues are rarely monitored within wild biota, leading to a knowledge gap in the extent and route of exposure these organisms encounter over their lifetime within their respective habitats. Thus, measurement of pharmaceutical tissue concentrations in aquatic wildlife is increasingly important. The challenges in understanding potential environmental risks are exacerbated by a large disparity between laboratory and the field-derived bioconcentration data. Surface water drug concentration measurements are a useful alternative, and have been the focus to date, but represent only one single compartment. For example, partitioning to sediments also needs to be considered, especially for benthic-dwelling organisms (Gilroy et al., 2012). Significant spatial and temporal fluctuations also exist (Miller et al., 2015; Luo et al., 2014). For pharmaceuticals, further complexity is added by their ionisation state in comparison to typical non-polar compounds, and this is important because it makes comparisons difficult across scenarios where water chemistry can impact ionisation and therefore uptake into biota (Karlsson et al., 2017).

Pharmaceuticals are often designed to cross biological membranes and therefore rate of uptake and internal concentrations are critically important. Therefore, to fully understand the potential for pharmaceuticals to cause harm in the aquatic environment, it is essential to assess wider occurrence in biota (including fish, invertebrates, plants and algae). The limited number of reports detailing occurrence in biota is potentially caused by two factors. The first of these is biological variation (there are estimated to be ∼31,000 fish species and ∼176,000 aquatic invertebrates described to date). The second is the analytical capabilities required for broad scope, multi-residue determination of thousands of human and veterinary pharmaceuticals and their metabolites in so many complex matrices at very high sensitivity.

The aim of this work is to review the occurrence of pharmaceutical residues in aquatic fauna. As part of this, a critical discussion will be presented focussing on (a) the range and reliability of analytical approaches for trace pharmaceutical and metabolite determination in aquatic fauna; (b) the reported occurrence of pharmaceuticals across a range of species, including fish and invertebrates, up to 2016; and (c) the bioaccumulation potential of pharmaceuticals and comparisons of field- and laboratory-based measurements. The use and collation of biomonitoring data to characterise pharmaceutical contamination is critical to understanding the extent of exposure and potential impact on aquatic fauna.

## 2 Systematic literature searching and statistical tests

A systematic search of published reports in the literature was performed using Scopus^®^ (Elsevier, Netherlands). Several keywords were included to identify published works for pharmaceutical occurrence in fish and invertebrates. These included “occurrence”, “PPCPs” or “pharmaceuticals”, and “fish” or “invertebrates”. The terms were searched across document titles, types, abstracts and keyword lists across all years up to 2016. The same keywords were also included in searches using Google Scholar up to 2016, to improve coverage of the available literature. Using this structured search strategy and to the best of our ability, all papers on pharmaceutical occurrence in aquatic fauna (fish or invertebrates) have been included, see Supplementary Information (SI) for full occurrence data tables. All statistical tests were performed in Minitab 18 (Minitab Inc., US) or Sigma Plot (Systat Software Inc., US) with a significance level set to alpha = 0.05.

## 3 Pre-treatment methods for pharmaceutical residues in fish and invertebrates

**Fig. 1:**
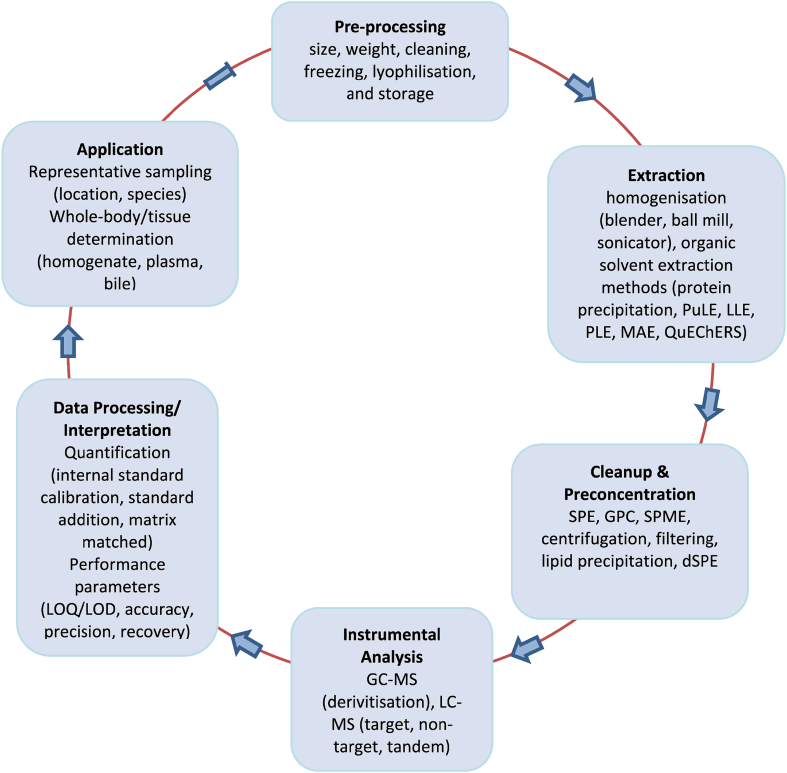
Overview of the analytical workflow for determination of exogenous or endogenous compounds from biological samples. The hyphenated arrow indicates that the work-flow can terminate after the application stage.

The typical workflow for determination of pharmaceuticals in abiotic or biota samples encompasses four overarching steps: sample preparation, confirmatory-level instrumental analysis data processing/interpretation and application (Fig. 1). Sample preparation can be further divided into sample collection/pre-processing, analyte extraction and clean up/pre-concentration. Analyte physicochemical properties have often governed the development and performance of robust analytical methods. Generally, studies focussing on biomonitoring cover small groups of compounds to maximise the quality of analytical determinations at high sensitivity. For analysis, either liquid (LC) or gas chromatography (GC) coupled to mass spectrometry (MS) are normally applied, due to their sensitivity and suitability for qualitative and quantitative analysis. For broad-scope quantitative screening, compound diversity can make analytical performance more variable across a wider analyte set.

Analytical methods vary for analysis of different biotic matrices, but mainly in their sample pre-treatment steps (Table S2 & S3) and special focus will be placed here in this review. It is important for broad-scope extraction methods applied to several thousand potentially relevant compounds, that a suitable extraction method is used to capture as many of these as possible, but while also limiting matrix interference. For pharmaceuticals by design, they generally follow Lipinski's ‘Rule of 5’ (Lipinski et al., 1997) defined as (a) small molecules (<500 Da, with the obvious exception of biopharmaceuticals, which for the purposes of this review, are excluded), (b) display octanol-water partition coefficients (logP) < 5 (in contrast to persistent organic pollutants, they are therefore of moderate polarity), and (c) have less than five proton donors and acceptors (and are therefore acidic, basic, zwitterionic and neutral molecules at physiological pH). These rules allow pharmaceuticals to partition better across membranes and are easily absorbed by the body (and by biota) as a result. As many are small molecules, they can generally be extracted from matrix by size relatively easily. Secondly, on an intra-molecular level they often contain either aromatic functionalities (which can be extracted via induced-dipoles or via *pi-pi* interactions), polar groups (e.g. amides, ketones, hydroxyl groups etc. which can be extracted via dipole-dipole interactions and/or H-bonding) and/or are often ionisable (extractable via ion exchange mechanisms). Pharmaceutical metabolites are usually more polar and exist either as transformation products or as conjugated substances. However, this is sometimes the opposite for pro-drugs, which may become less polar once bioactivated in the body. Recoveries and sensitivity for hundreds of compounds (precursors and biotransformation products) in a single assay are likely to vary substantially, as the ability to extract and detect many of them simultaneously is extremely challenging. As such, reliable measurement at the ng-μg g^−1^ level is mainly dependent on analyte recovery and matrix interference (both during extraction and instrumental analysis). In particular, sample size, matrix constituents/type, solvent chemistry (including pH, volume, salt/buffer/ligand concentration etc.), analyte chemistry, and operating conditions (temperature, agitation etc.) will affect the performance of any of the extraction techniques outlined above.

### 3.1 Biological fluids

In much the same way as for human body fluid matrices, liquid matrices from aquatic biota (such as bile or plasma) are commonly extracted via filtration, protein precipitation, centrifugation, followed by matrix clean up/analyte concentration. Solid phase extraction (SPE) has been used extensively for the latter and has enabled better selectivity and sensitivity for environmentally relevant pharmaceutical residue concentrations. Mixed-mode sorbents containing vinylbenzene co-polymerised or functionalised with polar moieties (e.g. N-vinylpyrrolidone, hydroxyl or cyano groups) or ion exchangers have dominated this field because they are more broadly applicable to moderate polarity and/or ionic compounds like many pharmaceuticals. A key drawback to this technique is that one sorbent may not provide sufficient recovery of all relevant compounds (for example very polar, conjugated metabolites and/or transformation products). As such, even with state-of-the-art analytical instruments, some compounds remain undetectable in extracts. However, with rapid advancements in instrumental sensitivity and chromatography techniques, direct analysis is becoming possible which may reduce this need for analyte concentration with SPE (Bayen et al., 2015; Campbell et al., 2011; Yu et al., 2012; Vergeynst et al., 2014). Focus may shift to using SPE only for matrix removal, if at all.

### 3.2 Solid and semi-solid matrices

**Fig. 2:**
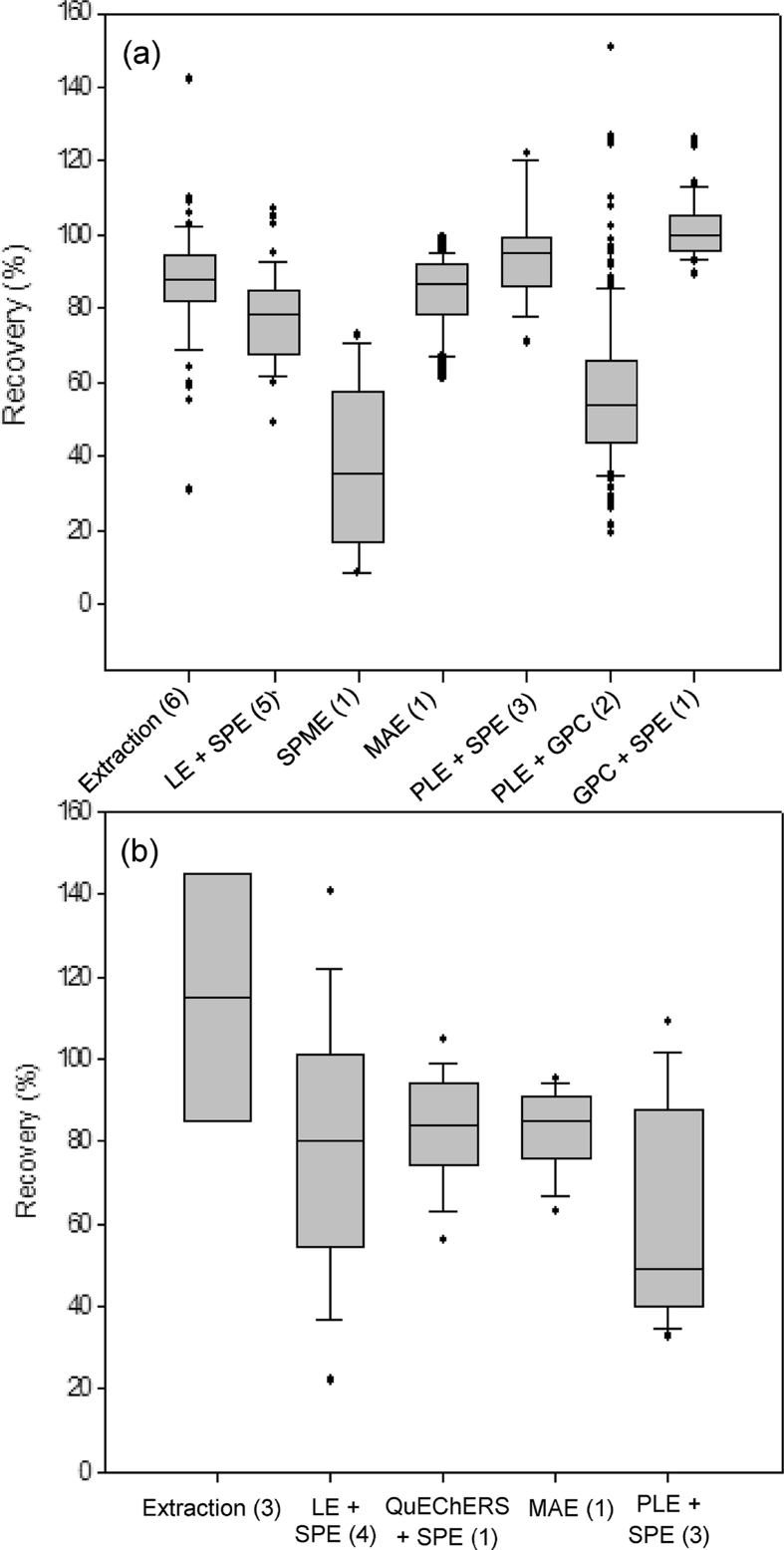
Comparison of reported recoveries for pharmaceuticals using different sample preparation methods determined in (a) fish and (b) invertebrates. Extraction indicates the use of liquid extraction only followed by either centrifugation and/or filtration. Boxes represent the 25th, median and 75th percentile, whiskers represent 10 and 90th percentile and dots indicate outliers. Parentheses indicate the number of studies for each method.

Clear mechanisms that govern occurrence in biota are lacking and this makes successful analyte pre-selection for method development much more difficult. The most common approach for sensitive analysis of semi-solid biotic matrices is the employment of two pre-treatment steps: (a) extraction of analytes from the solid matrix and (b) further matrix clean-up and analyte concentration to enable highly sensitive determination. Interestingly and importantly, while some niche applications may require it, increasing the sample preparation complexity for solids using several steps does not necessarily demonstrate improved method performance for small sets of individual compounds and where sensitivity may not be a significant hindrance. For example, Subedi et al. (2012) used a simple liquid extraction (LE) followed by evaporation of the extract and reconstitution in starting mobile phase for 17 pharmaceuticals and achieved recoveries ranging from 80 to 98%, with good precision. Several other works presented in Table S2-S3 have also simply used LE followed by centrifugation and/or syringe filtering have achieved similar recoveries across fish matrices (median recovery = 88%) (Subedi et al., 2012; Du et al., 2012; Ramirez et al, 2007, 2009; Schultz et al., 2010), compared to those employing increasingly complex sample preparations (Fig. 2(a)), such as LE and SPE (median recovery = 87%) (Brooks et al., 2005; Brown et al., 2007; Schuetze et al., 2008; Yu and Wu, 2015; Zhao et al., 2015), microwave assisted extraction (median recovery = 87%) (Fernandez-Torres et al., 2011), pressurised liquid extraction (PLE) followed by gel permeation chromatography (GPC) (median recovery = 52%) (Valdés et al., 2016). However, PLE and SPE together achieved the best recoveries with a median of 95% (Chu and Metcalfe, 2007; Li et al., 2012; Xie et al., 2015). PLE has also been used regularly on several abiotic samples (Jacobsen et al., 2004; Herrero et al., 2013; Krogh et al., 2008; Rodil and Moeder, 2008). In addition, PLE has advantages in that it is automated, can minimise thermal transformation of some labile pharmaceuticals, lower the solvent viscosity, improve analyte solubility, increase disruption of matrix-analyte interactions and increase diffusion rates enabling better extraction efficiency and recovery in a minimised extract volume (Barron et al., 2008). Only a single method used GPC together with SPE, where recovery was determined for a total of 13 pharmaceuticals (Tanoue et al., 2014). Only one study used solid phase micro extraction (SPME), but it showed the lowest recoveries from fish bile for a small set of six compounds (Togunde et al., 2012).

A newer, potentially more streamlined, broadly applicable extraction approach was introduced in 2003 termed Quick, Easy, Cheap, Effective, Rugged and Safe (QuEChERS) (Anastassiades et al., 2003), which has been widely adopted for pesticide residues and, more recently, pharmaceuticals and their metabolites in food (Berendsen et al., 2013; Lopes et al., 2012; Wiest et al., 2011) and environmental biota (Wiest et al., 2011; Daniele et al., 2016; Baduel et al., 2015). A set ‘cocktails’ of solvents, salts, ligands and buffers are added to an extraction mixture to broadly extract organic residues from (semi-) solids. For quantitation, precision can vary on a compound by compound basis, but it is very suited to broad-scope screening of a wider range of low-high polarity components. In general, salt choice and its influence on recovery tends to be compound and matrix dependent and this is mainly governed by solubility of each component in high/low ionic strength solutions. For example, atenolol showed MS signal enhancement in extracts of one species and suppression in another, using the same salts (Berlioz-Barbier et al., 2014). The addition of chelating ligands such as ethylenediaminetetraacetic acid (EDTA) can also improve extraction efficiency of some compounds which readily form complexes with matrices containing metals (Serra-Compte et al., 2017; Dasenaki and Thomaidis, 2015). Therefore, analytical methods that have been developed using one matrix may not show the same performance for another and methods need to be assessed carefully before application. For added specificity and to improve quantitative performance, QuEChERS is often combined with either SPE or dispersive SPE (dSPE) for matrix removal (Wiest et al., 2011; Cerqueira et al., 2014; De Carlo et al., 2015; Peysson and Vulliet, 2013; Huerta et al., 2013). Huerta et al. (2015) compared three sorbents for removal of invertebrate matrix and showed that a protein/phospholipid selective sorbent (Waters Ostro^™^) showed the best clean up efficiencies followed by a divinylbenzene-N-vinylpyrrolidone copolymer (Oasis HLB^™^) followed by magnesium silicate (Florisil^®^) for analyte recovery. Berlioz-Barbier et al. (2014) showed that use of primary-secondary amine (PSA) or PSA/C18 as dSPE sorbents significantly reduced analyte recovery for 28 out of 35 emerging contaminants (PPCPs, alkylphenols and plasticisers). Thus, purification was achieved with liquid-liquid extraction (LLE) using hexane as an alternative. In another study, a comparison of PLE, QuEChERS or ultrasonic extraction showed that overall PLE was superior to the other extraction techniques (Huerta et al., 2013). Furthermore, GPC and two types of SPE (Oasis HLB^™^ and Florisil^®^) were compared, with GPC showing greater performance in terms of recovery.

## 4 Matrix effects and analytical method validation

Overall, it is important to note that whilst one method may offer excellent performance for a single matrix, it might not perform as well for other compounds, or in other matrices, biological material, or species. A systematic approach to optimising workflows incorporating different extraction and clean-up workflows is critical, especially for pharmaceutical residue methods that require very high sensitivity across matrices. For example, fewer sample preparation approaches have been successfully optimised for pharmaceuticals in invertebrates when compared to fish. One observation is that the variance in recovery from invertebrates has generally been much greater for methods involving extraction followed by centrifugation, extraction followed by SPE and PLE and SPE (Fig. 2(b)). This can be partially explained by the use of smaller sample sizes of some invertebrates. In contrast to fish, analyses of invertebrate fluids such as haemolymph have only been reported for those dosed at high pharmaceutical concentrations (Gómez-Jimenez et al., 2008). As such for invertebrates, the most practical approach has been via whole-animal analysis. Inostroza et al. (2016) observed that an increasing sample size of *Gammarus pulex* (0.3–3 g) led to greater matrix effects and lower recoveries. However, no changes were observed above specific sample mass. We observed that scaling down the extraction procedure for *G. pulex*, including sample sizes from 100 mg to 50 mg, showed no statistically significant difference in mean recovery for ten selected pharmaceuticals (Miller et al., 2017). However, the precision (repeatability) of the method was significantly affected at smaller mass including added variability in matrix effects. Therefore, for small organisms there seems to be a critical and complex trade-off between sample size, recovery, matrix effects and precision in order to achieve optimised method sensitivity.

Several authors have (Ramirez et al., 2007; Berlioz-Barbier et al., 2014; Huerta et al., 2013) shown that both MS-related matrix effects and analyte recovery can depend on the extraction solvent used, where moderate polarity solvents enable better recovery and lower matrix effects. Virtually all analytical methods in fish and invertebrates either use methanol or acetonitrile as the organic extraction solvent (See SI, Table S2 & S3). One advantage of these moderately polar solvents is that lipids and proteins generally have low solubility in them and can therefore often be precipitated or partitioned away from the analytes of interest. For example, in fish, Huerta et al. (2013) found that lipid content caused MS signal suppression and enhancement. The highest signal suppression was found for fish livers which corresponded to the highest lipid content (48%), while whole fish homogenates had lower lipid content (15%). High signal suppression was also observed in a study across three marine mussel species where matrix effects were most significant for *Mytilus galloprovincialis* (lipid content 8.8%), in contrast to *Chamelea gallina* and *Crassostrea gigas* (lipid content 4.8% and 2.1%, respectively) (Alvarez-Muñoz et al., 2015). Matrix effects in relation to lipids have been well studied and have been noted to cause suppression or enhancement of the MS signal (Trufelli et al., 2011; Taylor, 2005).

In general, the mechanisms of MS-related effects are not yet fully understood, but are postulated to be related to several factors including (a) variable droplet formation efficiency and stability, (b) the limited numbers of excess charges and competition for space on the droplet surface, and (c) other physicochemical properties of the analyte, matrix and internal standard which may affect the ionisation process (e.g. polarity) (King et al., 2000; Bonfiglio et al., 1999; Matuszewski et al., 2003). In some cases, dilution can overcome matrix effects and even increase the sensitivity of an assay. Whilst focussing on pesticides in plants, Stahnke et al. for example, showed that dilution of extracts reduced ion suppression to ≤20% using a set of 156 analytes (Stahnke et al., 2012). However, given that matrix effects will still likely occur in most methods to some degree, the most reliable means to quantify pharmaceuticals using liquid chromatography-mass spectrometry (LC-MS) assays is to use stable isotope-labelled internal standards (SIL-IS) bearing ^13^C or deuterium atoms. Addition of SIL-IS(s) can improve linearity, accuracy and precision of an analytical method (Miller et al., 2017; Du et al., 2012; Benijts et al., 2004; Hartmann et al., 2007). Furthermore, whilst the SIL-IS can account for matrix effects on its unlabelled analogue, it may not do so for chemically different analytes and/or those that are chromatographically resolved (Huerta et al., 2013). Matrix variability can also affect accuracy of quantification (Wang et al., 2007; Jemal et al., 2003). The cost and availability of SIL-IS is also a limiting factor, especially for multi-residue analytical monitoring of metabolites/transformation products of interest in biota. Where SIL-IS are not used, then external matrix-matched calibration curves or standard addition calibration can be used. For the former, contamination with target analytes in the samples must be determined beforehand. For the latter, it is very time-consuming, requiring calibrants to be made for each sample and still may suffer from inhomogeneity in small solid sub-samples as well as variation of the native concentrations in collected wild organisms (i.e. background contamination).

Interestingly, of all the methods used to determine the occurrence of pharmaceuticals in aquatic environments, one study was found to follow method validation guidelines by the International Council for Harmonisation of Technical Requirements for Registration of Pharmaceuticals for Human Use (ICH) (Berlioz-Barbier et al., 2014). Many research works report “method performance” as a preliminary assessment of a freshly developed analytical method. Acceptance criteria are decided ultimately by the author which may only in part be based on recognised guidelines. (Miller et al., 2015; Du et al., 2012; Huerta et al, 2013, 2015; Alvarez-Muñoz et al., 2015). This is in stark contrast to other sectors where analytical workflows abide by very strict quality standards (Chandran and Singh, 2007). As environmental samples are often complex and vary between/within species, and even within biological compartments, recognised abiotic sample analysis validation guidelines may not yet be comprehensive enough to allow biomonitoring to be performed routinely. However, method validation guidelines used by the food science sector could be adopted here where, for example, the EU already has regulations concerning method validation in foodstuffs for consumer safety (EC and Commission Decision, 2002) and has been regulated since 1990 (EC, 1990).

## 5 Characterising the exposome with chromatography and high resolution accurate mass spectrometry (HRMS)

Compound mass analysis for biota samples has generally been performed with either triple quadrupole linear ion traps (QTrap) or triple quadrupoles given their selectivity, reproducibility and sensitivity in targeted multi-residue methods. Critical reviews of targeted quantitative analysis of pharmaceutical residues in such a complex array of different environmental/biological matrices have been reported in depth previously (Hernández et al., 2016). The focus here is more on methods that are applicable to even broader coverage of the exposome and the pharmaceutically-related fraction in particular. The advent of LC or GC coupled to high resolution mass spectrometry (HRMS) has made a significant impact in terms of more comprehensive characterisation of the exposome. Modern HRMS instruments include time-of-flight and Orbitrap mass analysers. The merits of each analyser type have also been reviewed extensively and is not the focus here (Nielen et al., 2007; Rousu et al., 2010; Eichhorn et al., 2012). However, the vast improvement in instrument mass accuracy and resolution has meant that shortlisting of suspect candidates for a given m/*z* is much more rapid and has significantly advanced the identification of new or emerging contaminants in the environment (Krauss et al., 2010).

Early stages of development of targeted methods may be open to analyte pre-selection bias where targets are chosen based on previously published investigations, leading to what is known as the ‘Matthew Effect’ (Daughton, 2014). Full scan HRMS however, enables a more comprehensive qualitative assessment underpinned by actual rather than presumed occurrence in biota. Broadly applicable methods can be developed covering many compounds having much wider chemical diversity. That said, previous occurrence data can also direct the post-hoc analysis of accrued data, but it does not preclude retrospective interpretation of additional data where needed.

To aid identification of non-target features, quantitative structure-retention relationship (QSRR) based tools have been developed for reliable *in silico* predictions for gradient LC retention time (Miller et al., 2013; Bade et al., 2015; Munro et al., 2015; Aalizadeh et al., 2016), even across different analytical systems (Barron and McEneff, 2016; Stanstrup et al., 2015). Such computational tools can add further assurance in feature annotation to help direct standard acquisition for unambiguous identification. For solid samples, the characterisation of the main biotransformation products of the antidepressant citalopram in sewage sludge were confirmed by incorporating this *in silico* prediction approach (Beretsou et al., 2016).

The main limitation of current LC-HRMS-based methods is the degree of coverage of the chromatographic separation space. That is, most occurrence data for emerging contaminants derive from separations performed using reversed-phase liquid chromatography (RPLC), which favours mid-non-polarity analytes. It fails for separation of very polar or inorganic compounds, although methods using ion chromatography (IC) coupled to electrospray ionisation (ESI)- or inductively coupled plasma (ICP)-MS (or MS/MS) analysers also exist (Barron and Gilchrist, 2014; Yuan et al., 2004). IC-HRMS have emerged for small organic and inorganic ions which are normally not retained well on RPLC stationary phases (Kohlmeyer et al., 2003). Furthermore, retention mechanisms in IC have been characterised to the point where *in silico* QSRR predictive tools can be used for tentative identification with excellent accuracy (Zakaria et al, 2009, 2010). However, to our knowledge IC-HRMS methods have not yet been widely applied to the analysis of biota, perhaps largely due to the rise in popularity of hydrophilic interaction liquid chromatography (HILIC) separation. HILIC is an adaptation of normal phase chromatography which can separate organic, inorganic compounds and ionised compounds with different selectivity to RPLC and IC. Prediction of HILIC retention times has recently emerged using machine learning (Taraji et al, 2017a, 2017b). Taken together, HILIC-HRMS is a promising tool for exposome characterisation.

In the context of this review, using LC-, GC- or IC-HRMS datasets combined with predictive tools for suspect identification in biological samples is especially advantageous as it enables the elucidation of biotransformation products and pathways, demonstrating the presence of conserved metabolic enzymes across species (Bletsou et al., 2015a). Furthermore, animals in the environment are exposed to a complex mixture of contaminants across their life cycle. To the authors' knowledge, only one untargeted HRMS profiling method has been applied to occurrence studies in environmental biota, tentatively identifying compounds using accurate mass measurements and isotope ratios of chlorinated or brominated compounds (Inostroza et al., 2016). Screening methods for foodstuffs using HRMS have allowed the qualitative determination of several hundred compounds including pharmaceuticals, pesticides and mycotoxins among others (Dasenaki and Thomaidis, 2015; Pérez-Ortega et al., 2016).

### 5.1 Application of HRMS for environmental metabolomics

In addition to screening for small micro-contaminant molecules, screening of biota for thousands of endogenous metabolites can be achieved simultaneously (i.e. metabolomics) (Gómez-Canela et al., 2016; Toyota et al., 2016a; Viant and Sommer, 2013). Environmental metabolomics-based studies have focused on the effect of biotic and abiotic stressors on both terrestrial and aquatic organisms and several have used nuclear magnetic resonance spectroscopy (NMR) and/or GC/LC-MS (Broeckling et al., 2005; Bundy et al., 2004; Bussell et al., 2008; Griffin et al., 2000; Hines et al., 2007; Skelton et al., 2014). For example, and in the context of the aquatic environment, Toyota and co-workers (Toyota et al., 2016b) recently used Fourier transform-ion cyclotron resonance direct infusion MS metabolite profiling to better understand the triggers and pathways governing the sex of offspring from *Daphnia pulex*. Jones and colleagues (Jones et al., 2008) applied ^1^H NMR and GC-MS to determine 32 and 51 metabolites, respectively, in order to identify pyrene exposure in the earthworm *Lumbriculus rubellus*. Southam and colleagues (Southam et al., 2014) used direct infusion MS to determine xenobiotic contamination of roach testes as a result of wastewater contamination and that the presence of three specific compounds (triclosan, chlorophene and chloroxylenol) themselves had a significant contribution to total wastewater induced biochemical changes.

Despite the breadth of metabolomics-based applications, reports on specific metabolic changes in aquatic organisms in response to emerging organic contaminants remain sparse. Moreover, little knowledge exists on the effects of sub-lethal concentrations of contaminants on aquatic biota and this is where environmental metabolomics could prove very useful. This is important to consider, as exemplified by the study of Álvarez-Muñoz et al. (2014) where LC-HRMS was used to examine the potential effect of alcohol polyethoxylates on *Solea senegalensis*. It was found that, despite being eliminated during depuration, this surfactant induced significant metabolite changes upon exposure, including a ∼106-fold increase of in circulating concentrations of C24 bile acids and C27 bile alcohols. Furthermore, glucocorticoid and lipid metabolism was disrupted and a 470-fold decrease in palmitoyl carnitine concentrations were observed (used in fatty acid transport). We also recently studied sub-lethal concentration exposures of three pharmaceutical compounds (propranolol, triclosan and nimesulide) to *G. pulex* (Gómez-Canela et al., 2016). Metabolic changes in a range of amino acids and other metabolites were identified and quantified using SIL-IS. In the real world, metabolic profiling of organisms is likely to be extremely useful for understanding non-lethal responses to environmental contaminants and could be used to elucidate metabolic pathways involved with such responses. David et al. recently studied the exposome and metabolome of roach which had been exposed to wastewater effluent (David et al., 2017). Exposure to effluent for 15 days resulted in large decreases on prostaglandin in tissue and in plasma among other perturbations in lipid metabolism. They concluded that whilst effects could be measured using ‘omics based approaches, identification of the array of contaminants causing such effects warrants further investigation, potentially by combining the broadly applicable screening methods highlighted earlier, and even by using the same analytical technologies (e.g. LC-HRMS).

As a last consideration using HRMS technologies and methods, linking observed phenotypic changes resulting from pharmaceutical exposure to individual metabolic profiles and internalised drug concentrations would potentially enable a powerful and mechanistic approach to the assessment of stressors at environmentally relevant concentrations.

## 6 Pharmaceuticals in the aquatic environment

Pharmaceutical occurrence in abiotic environmental compartments has been the primary focus of monitoring research over the past two decades (Daughton, 2016). A recent publication has compiled a database of 123,761 entries for 631 unique pharmaceuticals reported across 71 countries (aus der Beek et al., 2016). Only 16 pharmaceuticals have been found in all UN regions indicating that there is regional variability between occurrence. Furthermore, there are significant regional and national differences in the prescribing of specific drugs and brands. This leads to variation in the source of pharmaceuticals entering the environment via patient use different from one country to the next, and the resulting environmental concentration then further depends on the extent of sewage effluent treatment, and local as well as regionally variable water availability. However, the wealth of data for abiotic compartments cannot be extended to the biotic compartments. The number of articles reporting pharmaceutical occurrence in biota is relatively scarce, with only 43 publications (covering 18 countries, see SI) in comparison to water/sediment compartments with a total of 1166 publications (aus der Beek et al., 2016) at the time of writing this review. Nevertheless, over the last decade the number of reports that have determined the occurrence of pharmaceuticals in aquatic biota has increased significantly. Fish occurrence has been reported from over a longer period and in marginally greater numbers (29 publications) than for invertebrates (18 publications).

### 6.1 Regional occurrence of pharmaceuticals

The spatial distribution of monitoring campaigns also differs between geopolitical regions (See SI, Figure S1). In North America and Europe, there is a predominance of occurrence data for fish and invertebrates, respectively. East Asia, has had relatively few studies with the focus on occurrence within fish. The only occurrence study in South America was in Argentina performed with fish homogenates (Valdés et al., 2014). Across fish species, 179 measured pharmaceutical concentrations have been reported in China (across 5 publications) and 155 measured values were also reported from locations in the U.S. (across 10 publications). These two countries account for 68.2% of the positive quantifications determined in fish. Measured concentrations from China covered predominantly antibiotic classes whereas in the US they covered antidepressants, antihistamines and calcium channel blockers. The focus of antibiotics by China has been reported previously for surface waters (aus der Beek et al., 2016). Similarly, for the invertebrates, measured concentrations are predominantly from the US (65 measurements, across 5 publications) and China (63 measurements, across 2 publications) forming 64% of the measured concentrations.

There are no reported data on pharmaceuticals in biota from Eurasia (i.e. Russia), Africa, and Australia. These regions also have very few reported pharmaceutical occurrence data for any compartment of the aquatic environment (e.g. sediment, water etc.), thus the potential impacts of these contaminants within these regions are not well characterised (aus der Beek et al., 2016). This could be particularly important for developing regions (Tijani et al., 2016) that have heavily industrialised areas used for pharmaceutical production (Joakim Larsson and Fick, 2009). As an example, exceedingly high concentrations of pharmaceuticals have been determined from manufacturing effluents in India (Larsson et al., 2007), but to the authors’ knowledge no occurrence in biota has been reported from the region. Occurrence studies from African countries such as Kenya (Prasse et al., 2010), Nigeria (Olarinmoye et al., 2016) and South Africa (Matongo et al., 2015) have shown surface and wastewater pharmaceutical concentrations in the range generally found in other regions (ng to μg L^−1^). Anti-retrovirals which are often not studied in other regions accounted for the largest portion of the total pharmaceutical loads in the measured surface waters (Prasse et al., 2010). In Australia, pharmaceuticals such as antibiotics, non-steroidal anti-inflammatory drugs (NSAIDs), antidepressants and lipid regulators have been detected ranging from ng L^−1^ - μg L^−1^ in surface waters and waste waters (Scott et al., 2014; Watkinson et al., 2007; Hashim and Khan, 2011). Scott et al. (2014) reported that monitoring of 73 freshwater sampling sites revealed that carbamazepine and sulfamethoxazole had hazard quotients >1 indicating the possibility for adverse effects.

Overall, and given the above examples, international differences in pharmaceutical occurrence arguably highlights a need to understand the scale of geopolitical influence better. Targeted biomonitoring programmes for certain classes of compounds will likely fail to capture the breadth of the issue and until more countries engage with biomonitoring, understanding the risk of pharmaceutical exposure in non-target biota is limited. It is the internal exposure which will likely drive impact rather than the water concentration alone.

### 6.2 Prevalence of therapeutic classes determined in fish and invertebrates

Within the fish monitoring studies, a total of 490 positive quantifications have been reported in the literature, which cover 31 different therapeutic classes of pharmaceuticals. Of these a total of 35% were antibiotics, 18% were antidepressants, 11% were NSAIDs, 10% were antihistamines and the remaining 23% of determinations were from various pharmaceutical classes (Figure S1). In contrast, quantification of 200 pharmaceuticals have been reported within the aquatic invertebrate phylum covering 27 classes. A total of 34% were reported as antibiotics, 22% were antidepressants, 14% were NSAIDs and the remaining 30% were attributed to various other classes. In both fish and invertebrates, the majority of reported internal concentrations are covered by the antibiotic and antidepressant classes. This will be in part due to consumption and usage patterns where high consumption may lead to higher input in the environment. For example, antidepressant usage has increased in Europe by 20% from 2000 to 2010 (Gusmão et al., 2013) and use has approximately increased by two-fold among OECD countries since 2000 (OECD, 2015). Similarly in the US antidepressant usage has increased significantly and these are the third most prescribed drug across all age groups (Pratt et al., 2005). However, prescription and over-the-counter medication use can vary by concentration administered and duration of the course/use/season. Therefore, prescription rates alone may not explain detection patterns in the field, particularly as dilution is so variable and can be as much as four orders of magnitude (Keller et al., 2014). This is further highlighted with the lipid regulators that have high consumption rates (OECD, 2015) yet have accounted for 1.5% of the determinations in fish and 0.5% of the determinations in invertebrates.

The coverage of pharmaceutical classes reported can also be explained by several other factors such as seasonal trends, removal efficiency by WWTPs and the bioavailability of the compound. The absence of certain compounds may also be associated with the aforementioned Matthew Effect (Daughton, 2014) which limits our characterisation of the exposome and the potential risk of other compounds. Consumption data and usage trends could be, in part, used to direct targeted lists of compounds for biomonitoring as is the case in water-based monitoring studies. For example, carbapenems and polymixins have been increasing in their usage (Van Boeckel et al., 2014), which are classes that are not often monitored for (as with other classes such as anti-retrovirals). In addition, it is also important to look at the spatial occurrence including coverage of therapeutic classes together with the regional consumption patterns as it may aid in the identification of potential high-risk areas where there is a lack of measured data. Antibiotic consumption from 2000 to 2010 has been reported to have increased by 36%, with 76% of this increase resulting from use in Brazil, Russia, India, China and South Africa (Van Boeckel et al., 2014). Thus, surveillance of these regions for these classes of compounds will be important for identifying any potential unacceptable risk. The occurrence of antibiotics in the environment is also a human health hazard due to the rise of antimicrobial resistance (Hirsch et al., 1999). The occurrence of trace pharmaceuticals in animals (and other environmental compartments) could drive selection pressures for mobile genetic elements associated with drug-resistance (Wellington et al., 2013). Thus, measuring antibiotic occurrence in biota could also aid the surveillance of antimicrobial resistance (Le Page et al., 2017).

## 7 Occurrence of pharmaceuticals in fish species

At present, a total of 61 different species of fish have been used to study the occurrence of pharmaceuticals across both freshwater and marine environments. Comparison of the reported fish tissue and plasma/bile concentrations showed quite clearly that fish tissue concentrations had much greater coverage. This is perhaps surprising given that plasma measurements can be useful for determining potential effects when related to the human therapeutic plasma concentrations (C_max_) by means of the Fish Plasma Model (Huggett et al., 2003). However, the authors note that there can be significant difficulty in obtaining plasma samples from fish in the field where whole-body and tissues samples allow much greater flexibility, especially for animals that are small in size (particularly for haemolymph sampling in invertebrates). The use of the Fish Plasma Model has been investigated for more than 10 years (Rand-Weaver et al., 2013) and recently direct evidence of cause and effect has been established (Margiotta-Casaluci et al, 2014, 2016). Thus, the use of this model would complement biomonitoring studies as it would allow the derivation of the effect ratio (ER) and hence the potential risk to fish populations. The model was field tested using sewage effluents by Brown et al. (2007) and Fick et al. (2010) where several pharmaceuticals achieved a comparable plasma concentration to human therapeutic levels, with ERs ranging from <1 to >1000.

**Fig. 3:**
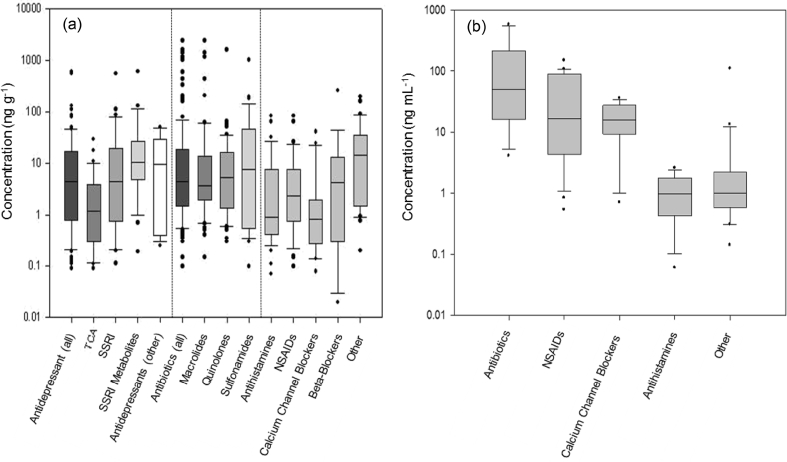
Reported concentrations of different pharmaceutical classes determined in fish (a) tissues and (b) plasma or bile. The Other category denotes various pharmaceutical classes that were reported infrequently from the literature. Boxes represent the 25th, median and 75th percentile, whiskers represent 10 and 90^th^ percentile and dots indicate outliers.

The determination of internal concentrations in fish species has ranged from 0.02 to 2390 ng g^−1^ across tissues and from 0.055 to 567 ng mL^−1^ in fish plasma or bile measurements (Fig. 3 and Table S2). The highest concentrations reported in fish tissue stem from the macrolide/quinolone antibiotics and in fish plasma/bile measurements. Erythromycin was measured at 545 ng mL^−1^ and lincomycin was measured at 567 ng mL^−1^ in plasma. Sulfonamides and quinolones have been quantified in plasma, however, remained ≤144 ng mL^−1^ (sulfamethazine). NSAIDs and calcium channel blockers are the next highest determined concentrations in fish bile and plasma measurements with medians of 16.5 ng mL^−1^ and 15.74 ng mL^−1^, respectively. Antihistamines and other pharmaceutical classes (including tricyclic antidepressants (TCA), lipid regulators, selective serotonin reuptake inhibitors (SSRI) and antipruritics (among others)) have been reported with median concentrations 0.97 and 1 ng mL^−1^, respectively. The relatively lower concentration of these compounds could be explained by tissue specific accumulation (i.e. tissue partitioning > blood partitioning), or that the compounds show relatively little accumulation overall. For example, the antihistamine tissue concentrations had a median of 0.91 ng g^−1^ which corresponds well with the median values determined for the plasma/bile median, suggesting that the antihistamine compounds that have been measured potentially have low accumulation in fish. An important metric used regularly in pharmacology that could inform the potential for tissue distribution of pharmaceuticals in non-target biota is volume of distribution (V_D_), where databases are available with this information summarised (Berninger et al., 2016).

### 7.1 Antidepressants and their biotransformation products determined in fish

Antidepressant measurements within fish tissues ranged up to 600 ng g^−1^, with the largest concentration measured within the SSRI metabolites (norsertraline) (Du et al., 2012). The TCAs have been reported at lower concentrations in comparison to other antidepressant classes. The 75th percentile of occurrence data was 3.51 ng g^−1^, ∼5–9-fold lower than the 75th percentile of the remaining classes. Whilst not strictly a TCA, most frequently reported tricyclic pharmaceutical was carbamazepine, which has also been reported to have low bioaccumulation factors in several species (Miller et al, 2016, 2017; Meredith-Williams et al., 2012; Boillot et al., 2015; Garcia et al., 2012). Other antidepressants including the serotonin–norepinephrine reuptake inhibitors (SNRI), norepinephrine–dopamine reuptake inhibitor (NDRI) and benzodiazepines were reported to occur at similar concentration ranges to the SSRI class. Interestingly, within the SSRI class of pharmaceuticals, the spread of the metabolite data showed larger summary statistics (mean, median, min/max and interquartile ranges) indicating that SSRI metabolites were often present at higher concentrations in comparison to the precursor compound. This suggests that SSRI metabolites may be more accumulative than their precursor, or that metabolism is rapid. This higher accumulation has been observed from individual studies where Du et al. (2012) measured much higher relative concentrations of the SSRI metabolite norsertraline in both liver and muscle tissue. This is also consistent with toxicokinetic studies where some other classes of pharmaceutical metabolites have accumulated more than the parent compounds (e.g. benzodiazepines) (Miller et al., 2017; Lahti et al., 2011). A *t*-test assuming unequal variances showed that the there was no statistically significant difference between the reported concentrations of either group (*p*-value 0.67, alpha = 0.05). Nonetheless, if some biotransformed products do have higher accumulation than precursor compounds, then there is a potential for greater risk in the environment. Prodrugs are an important consideration here, as the biotransformation product is the pharmaceutically active compound. However, as biotransformation is often not considered during monitoring campaigns, and only in part of the European regulatory environmental risk assessment since the focus is on the product, there remains a potential gap that should be addressed in future works (Boxall et al., 2012).

### 7.2 Antibiotics and other determined therapeutic classes in fish

Among antibiotic measurements the highest detected concentrations were 2390 ng g^−1^ for erythromycin (Zhao et al., 2015) and 1600 ng g^−1^ for ormetoprim (Meador et al., 2016). Whilst these compounds have been reported to reach these high concentrations, most often the concentrations remain relatively low. Median values for macrolides, quinolones and sulphonamides were 3.60, 5.23 and 7.35 ng g^−1^, respectively. The only other class of antibiotics to be quantified in fish were the tetracycline compounds, oxytetracycline (50 ng g^−1^) and chlortetracycline (160–590 ng g^−1^). Beta-lactam antibiotics have not been determined in fish (or invertebrates) although measured concentrations have been determined in surface waters ranging from low ng L^−1^ to mid ng L^−1^ concentrations (aus der Beek et al., 2016). The remaining classes of compounds including the antihistamines, NSAIDs, calcium channel blockers and beta-blockers showed relatively low concentration ranges when compared with the antidepressants or antibiotics, with the 75th percentile ranging from 1.71 ng g^−1^ (calcium channel blockers) to 11.51 ng g^−1^ (beta-blockers).

The majority of reported concentrations in each of these therapeutic classes is largely representative of only a single compound such as diclofenac (NSAIDs), diphenhydramine (antihistamines), propranolol (beta-blockers) and diltiazem (calcium channel blockers). These are often the most common compounds that are targeted and demonstrate the potential bias associated with the Matthew Effect. The data for these single compounds show that measured concentrations can vary quite considerably for the same compound (Figure S2). The scatter in the measured concentrations is likely to arise from the temporal and spatial fluctuations of surface water/sediment pharmaceutical concentrations in addition to other factors (pH, bioavailability, temperature etc.).

Diclofenac, in particular, was measured at concentrations reaching up to 148 ng mL^−1^ in plasma and 13.8 ng g^−1^ in fish muscle, from field samples. The concentration in the muscle tissue is approximately 5-fold lower than the concentration in the muscle associated with the No Observed Effect Concentration (NOEC) of 1 μg L^−1^ proposed by Schwaiger et al. in water (Schwaiger et al., 2004). However, the Predicted No Observed Effect Concentration (PNEC) has been proposed at 0.1 μg L^−1^ and measured environmental concentrations have exceeded this value in 12 countries (aus der Beek et al., 2016). Measured plasma concentrations of diclofenac exposed at 1.6 μg L^−1^ reached ∼9 ng mL^−1^ and exposure at 11.5 μg L^−1^ reached ∼60 ng mL^−1^ (Cuklev et al., 2011). In another study, plasma concentrations of diclofenac reached over 113.1 ng mL^−1^ and was associated with an exposure level of 5 μg L^−1^ over a 21-day period (Bickley et al., 2017). These reports indicate that concentrations of diclofenac in fish plasma from the field have exceeded laboratory determined plasma concentrations associated with exposure levels much greater than some proposed PNEC or NOEC. However, there remains an unresolved debate regarding the replicability of the reported ecotoxicology studies; some suggest the population endpoint relevance could be as much as 320 μg L^−1^ in the water (Memmert et al., 2013). The ongoing debate in the literature is critical to resolve the balance between protecting the environment from population adverse effects, and weighed against this the value of human medicines (Acuña et al., 2015).

The ‘other’ grouping of pharmaceuticals was combined measured data that comprised another 17 various pharmaceutical classes but had only been reported with a total of ≤7 measured values. Lipid regulators are one such class and have had only 7 reported occurrences across fish despite them being some of the most prescribed compounds globally (OECD, 2015; Lindsley, 2012). In liver tissue, gemfibrozil has been measured reaching up to 90 ng g^−1^ (Ramirez et al., 2009). The highest measured plasma concentration was 109 ng mL^−1^ (Brown et al., 2007). Plasma concentrations of 170 ± 20 ng mL^−1^ have been shown to be associated with a 50% decrease in goldfish testosterone levels, suggesting the possibility of endocrine disruption (Mimeault et al., 2005). However, the human therapeutic concentration is approximately 2500 ng mL^−1^ without changes in testosterone (Schulz et al., 2012). If gemfibrozil does elicit endocrine disruption in fish, the maximum measured environmental concentration (109 ng mL^−1^) leads to the possibility of adverse effects in the environment if fish are acutely susceptible to testosterone modulation. Currently, effects related to measured internal concentrations are limited. Effect-based studies have generally linked exposure concentrations to observed effects. Thus, the cause and effect relationship is limited as toxicological endpoints are inferred from a dosed concentration in water rather than using measured internal concentrations. The use of internal concentrations are critical as they can directly link the cause and effect leading to improved risk assessment (Rand-Weaver et al., 2013), yet it was only a few years ago that this was first demonstrated in fish (Fick et al., 2010).

## 8 Occurrence of pharmaceuticals in invertebrates

**Fig. 4:**
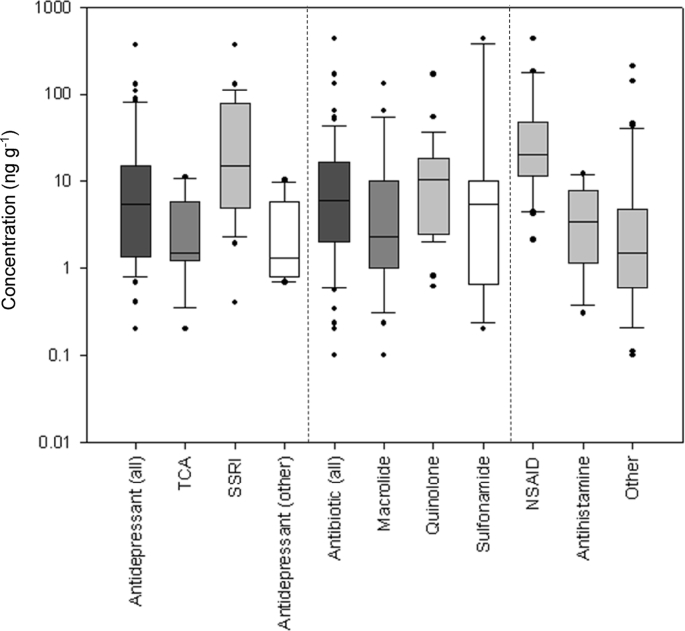
Reported concentrations of different pharmaceutical classes determined across various invertebrate species. The Other category denotes various pharmaceutical classes that were reported infrequently from the literature. Boxes represent the 25th, median and 75th percentile, whiskers represent 10 and 90th percentile and dots indicate outliers.

The most frequently reported concentrations in invertebrates belong to antibiotics and antidepressant therapeutic classes, with concentrations determined ranged from 0.20 to 320 ng g^−1^ for the antidepressants, 0.10–430 ng g^−1^ for antibiotics, 2.10–430 ng g^−1^ for NSAIDs, 0.30–12.10 ng g^−1^ for antihistamines and 0.10–210 ng g^−1^ for the other therapeutic classes (Fig. 4). Within the antidepressant class, the SSRIs showed relatively higher internal concentrations when compared to the TCAs and the other antidepressant classes including benzodiazepines and SNRIs. The higher occurrence of the SSRIs also compares with the fish occurrence data where SSRIs showed increased concentrations relative to the TCA class. Therefore, the data suggests that the SSRI class of antidepressants may have a higher risk potential than other antidepressants. This would reflect current trends in effects studies which have focussed on SSRI antidepressants (Silva et al., 2015). However, the most frequently reported SSRIs from biomonitoring studies are fluoxetine and sertraline that may lead to possibility of biasing occurrence data. Further, both of these drugs have complex pharmacology where the primary metabolites are the pharmacologically relevant compound, and transformation in the environment will play an important role in their risk profile. Whilst the data available suggests that individual compounds such as fluoxetine might have increased risk in the environment (Fong and Ford, 2014; Brooks, 2014), other studies have demonstrated responses only at non-environmentally relevant concentrations engineered to generate internal concentrations in the human therapeutic range (Margiotta-Casaluci et al., 2014).

### 8.1 Antibiotics in invertebrates

The maximum concentration determined among the antibiotic classes was from the compound sulfamethazine which reached up to 430 ng g^−1^ (Dodder et al., 2014). However, in general, the sulfonamides showed low level occurrence with all remaining measured concentrations <15.3 ng g^−1^. Toxicity of selected sulfonamide antibiotics (sulfamethazine included) have been reported in crustaceans with EC_50_ levels for often in the mg L^−1^ range (García-Galán et al., 2009; Białk-Bielińska et al., 2011; De Liguoro et al., 2009). Furthermore, hazard quotients have been estimated for fish and invertebrates and demonstrate that sulfonamides have low risk in the aquatic environment (with the exception to algae) (García-Galán et al., 2009). The macrolides and quinolones show maximum measured concentrations of 132 ng g^−1^ and 170 ng g^−1^, respectively. However, in general these classes of compounds showed low occurrence in invertebrates with median concentrations of 2.32 ng g^−1^ and 10.60 ng g^−1^. Available effect data for the quinolones is limited, but a study involving the toxicity of ciprofloxacin to fish and invertebrates showed toxicity thresholds of mg L^−1^ concentrations on a range of endpoints (mortality, growth, reproduction) (Martins et al., 2012). The paper showed that hazard quotients were <1 indicating little or no risk. (Martins et al., 2012; Halling-Sørensen et al., 2000). However, it should be noted that for antibiotic toxicity hazard quotients, the most sensitive endpoints (including luminescence inhibition and growth) determined across the therapeutic classes are usually algae and cyanobacteria (Yang et al., 2011; Ji et al., 2012; Baumann et al., 2015), thus these often represent the worst-case scenario (Le Page et al., 2017).

### 8.2 NSAIDs, antihistamines and biotransformation products determined in invertebrates

**Fig. 5:**
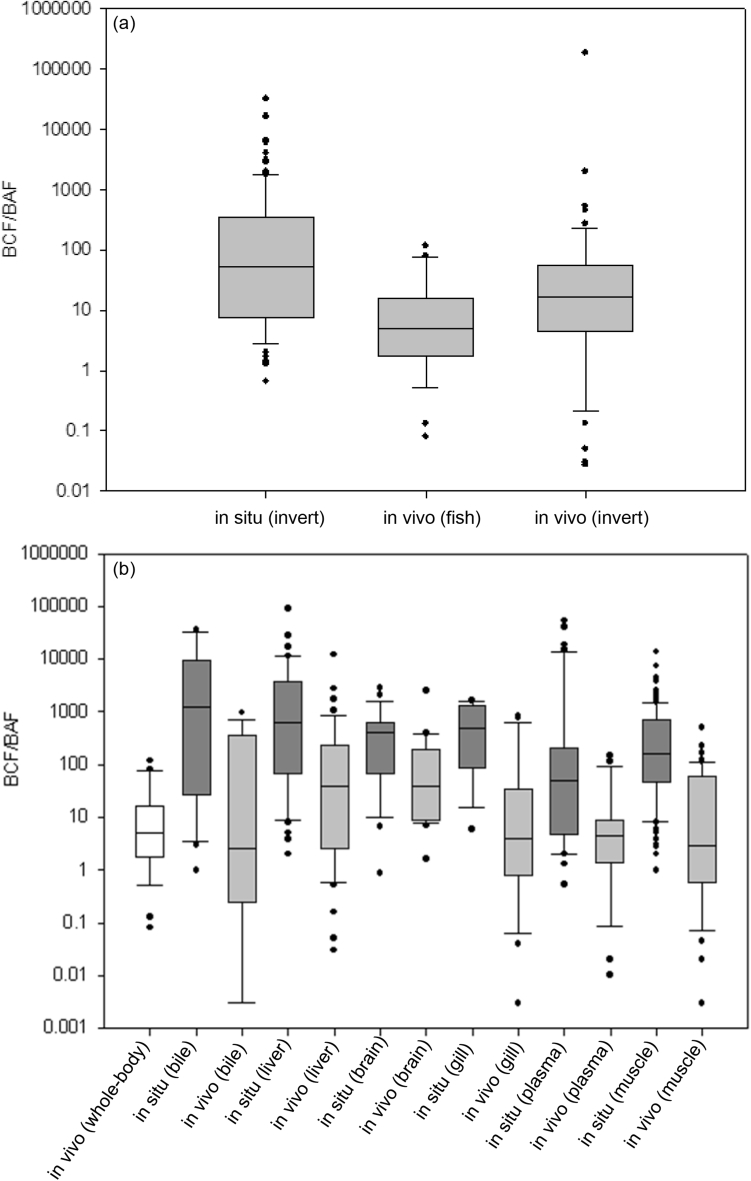
Reported accumulation of pharmaceuticals in fish and invertebrates determined in the field (*in situ*) and in the laboratory (*in vivo*). (a) comparison of reported whole body pharmaceutical accumulation in fish and invertebrates (invert) (b) comparison of reported pharmaceutical accumulation in different fish tissues determined in the laboratory and in the field. BCF/BAF axis label denotes that the value is either a BCF or a BAF. Boxes represent the 25th, median and 75th percentile, whiskers represent 10 and 90th percentile and dots indicate outliers.

The remaining most frequently detected compounds belonged to the class of NSAIDs and antihistamines. The antihistamine group consisted of only measurements of diphenhydramine which showed median concentrations of 3.38 ng g^−1^, with the largest measured internal concentration reaching 12.10 ng g^−1^. As with occurrence in fish, this suggests that the compound diphenhydramine has a relatively low accumulation potential (albeit several higher measurements were reported in fish, Table S2/S3). Alternatively, the low measured internal concentrations may be a result of a low occurrence in surface waters. NSAID residues in invertebrates were relatively higher (median = 20.50 ng g^−1^) than compared with other classes of pharmaceuticals (Fig. 5). The most frequent NSAID determined was diclofenac with a median of 15 ng g^−1^, followed by ibuprofen with a median of 83.65 ng g^−1^ and celecoxib with a median of 24 ng g^−1^. In general, while they show relatively higher measured internal concentrations, the risks NSAIDs pose to invertebrates have been reported to be low (Constantine and Huggett, 2010; González-Ortegón et al., 2013; Heckmann et al., 2007).

Biotransformation products have been determined relatively infrequently across invertebrates with only 12 reported concentrations (See SI, Table S3), half of which were associated with norsertraline. Such infrequency in occurrence reports can be explained in part by the low number of analytical methods that have targeted biotransformation products. Comparison of measured internal concentrations of sertraline and norsertraline showed that they were quantified in the same ranges (0.4–370 ng g^−1^ and 9.8–88 ng g^−1^, respectively). The remaining metabolites quantified included carbamazepine-10,11-epoxide, 2-hydroxycarbamazepine, 10-hydroxyamitriptyline, norfluoxetine and norvenlafaxine, all of which were ≤4.55 ng g^−1^, similar to measured concentrations of their precursors. Biotransformation product occurrence data are limited for surface waters and biota. However, untargeted analytical methods that can be used to profile extensive xenometabolic pathways of pharmaceuticals in biota are now emerging (Bletsou et al., 2015b; Rösch et al., 2016). These methods will thus be valuable for determining which biotransformation products to target going forward.

## 9 Field versus laboratory accumulation of pharmaceuticals

Whole body accumulation is expressed as either (a) bioconcentration factors (BCF) representing the compound accumulation solely from the water, or (b) bioaccumulation factors (BAF) that represent accumulation from both the water and diet. Field based assessment of accumulation is reported as BAF. A further term used in defining accumulation is a biomagnification factor (BMF), which describes the propensity of a compound to biomagnify up the food chain. Larger values of BCF, BAF or BMF indicate more pronounced accumulation where regulatory thresholds for compounds to be considered bioaccumulative have been defined as ≥1000 L kg^−1^ (depending on the regulatory body). It must be noted that BCF in the academic literature often is calculated as the ratio between the instantaneous measured internal and external concentrations i.e. a BCF of 10 would suggest that the internal concentration was found to be ten times that of the water. In a regulatory context, the BCF is a kinetic measure established via a standardised test protocol (e.g. OECD 305) where it is the ratio between the rate of uptake and the rate of depuration. Occurrence data can often indicate compounds that may show a potential to accumulate in organisms. However, it is laboratory *in vivo* exposures studies that can elucidate toxicokinetic/toxicodynamic (TKTD) properties of these compounds, identify hazards associated with exposure, and help prioritise compounds of concern for targeted analytical approaches in environmental monitoring campaigns. The earliest published laboratory study to determine a pharmaceutical bioconcentration factor that we could find was by Hou et al. (2003) in 2003, where the authors exposed sturgeon to sulfamethazine. Currently, 57 publications have dealt with the accumulation of pharmaceuticals across fish and invertebrates, with approximately >640 BCF/BAFs covering 90 unique pharmaceuticals estimated (field BAFs, *n* = 418). The most frequently reported BCF or BAF data included six pharmaceuticals in the order of: carbamazepine > erythromycin > diclofenac > roxithromycin > ibuprofen > propranolol. The compounds carbamazepine, propranolol, ibuprofen and diclofenac have also been reported to be the most the frequently cited compounds (among 8 others) from prioritisation reviews of emerging environmental contaminants (Donnachie et al., 2016).

Current evidence does not indicate that pharmaceuticals biomagnify (Du et al., 2014; Lagesson et al., 2016; Xie et al., 2017). In contrast, studies focussed on trophic transfer of pharmaceuticals have observed that internal concentrations are often larger in invertebrates when compared to fish. This suggests that dietary accumulation of some pharmaceuticals is likely to play a minor role in accumulation (Du et al., 2014; Lagesson et al., 2016), at least in fish. Comparison of whole body accumulation data in invertebrates showed that field determined values could be perceived as higher when compared to estimates from laboratory studies (Fig. 5(a)). A single factor ANOVA showed that there was no statistically significant difference between the field and laboratory values (*p*-value = 0.536). No statistically significant difference between the sensitivities to accumulation in invertebrates and fish were observed within the laboratory data (one tailed *t*-test assuming unequal variance, *p*-value = 0.155). However, it should be noted that the whole-body accumulation data are not homogenous, so differences in accumulation between the organisms for the same compounds might not be apparent. Furthermore, field BAFs for fish were limited with only one study published that determined a whole-body BAF (Valdés et al., 2016).

The largest laboratory BCF reported was for the SSRI fluoxetine that reached 185,900 L kg^−1^ in the freshwater arthropod, *Gammarus pulex* (Meredith-Williams et al., 2012). This compound has been the subject of many publications related to its impact in the environment for all persistence, bioaccumulation and toxicity (PBT) criteria (Brooks, 2014; Brooks et al., 2003). However, several other accumulation estimates were shown to be > 62-fold lower (Table S3), albeit for different animals under different experimental conditions. The largest measured BAF was for the compound hydroxyzine (96,000) in a freshwater snail (*Planorbidae* sp.) (Lagesson et al., 2016). However, this compound was shown to have a large accumulation range across different species (fish and invertebrates) with the lowest estimated BAF_muscle_ of 1050 for *Perca fluvitalis,* indicating that sensitivity to accumulation can vary across species by ∼100-fold. Hydroxyzine was also shown to have an *in vivo* BCF of 2000 in *Zygoptera* larvae that was approximately 7-fold lower than the *in situ* BAF determined for the same species (14,340) (Lagesson et al., 2016; Jonsson et al., 2014). Taken together, this suggests that this compound may be bioaccumulative, particularly to invertebrates. However, exceptionally high values of accumulation should be considered with caution, if correct we should expect to see this widely repeated in analysis of environmental samples in future.

The difference between laboratory and field estimations of accumulation could be attributed to dietary assimilation and other environmental/ecological factors such as feeding strategy, habitat choice, water chemistry and bioavailability (Vignati et al., 2007; Hird et al., 2016). As *in vivo* tests do not often include dietary exposure, it has been suggested that this may underestimate the accumulative potential of a compound (Lagesson et al., 2016). However, while dietary exposure can affect accumulation, studies have demonstrated that accumulation of contaminants through the diet generally show little influence on the compounds that have been tested and may only be more relevant for higher trophic levels (Lagesson et al., 2016; Ashauer et al., 2010; Du et al., 2015). Differences in accumulation have also been shown where multiple species have been analysed from the same field site (Li et al., 2012; Xie et al., 2015). The differences in accumulation here have been proposed to be influenced by a number of factors, including lipid content of the organism, body size, life stage and respiration strategy (Meredith-Williams et al., 2012; Arnot and Gobas, 2006; Ruhí et al., 2016; Rubach et al., 2010).

With this variety of different factors affecting accumulation, comparisons of BAFs/BCFs between publications may hold little to no value unless the same organism, location and indeed all experimental and analytical conditions are replicated (Xie et al., 2015). Additional reasons for the observed disparity between field and lab-based bioaccumulation data is due to the fluctuations in both spatial and temporal concentrations of pharmaceuticals in the field. This is the inherent uncontrolled variance that can be associated with *in situ* measurements (Xie et al., 2015; Scott et al., 2016; Cantwell et al., 2017). Therefore, field-derived BAF estimations using surface water concentrations may lead to over/underestimates. Additionally, it is difficult to characterise the exposure that animals have received, since many species are migratory (due to factors such predator avoidance, drift, seeking food or a mate, and seasonal environmental changes). However, there is still value in spot samples of measured internal concentrations in the field because they can give an integrated value of temporal exposure that include toxicokinetic/toxicodynamic processes. The fluctuation of the environmental exposure probably more closely relates to the changes in pharmacodynamics within man. It is the area under the curve (AUC) in the treatment scenario that is most important to the pharmacologist and the half-life of a drug within the patient is of critical value, but few ecotoxicology studies have attempted to address this. One exception looking at the effects of glucocorticoids linked the AUC to internal exposure resultant from a fluctuating water exposure concentration and used this to develop a quantitative adverse outcome pathway for this class (Margiotta-Casaluci et al., 2016). This concept could be the basis of future laboratory studies, in order to more realistically mimic the environment accounting for exposure fluctuations as long as dosing is measured appropriately.

### 9.1 Tissue specific accumulation of pharmaceuticals

The disparity between in-field and laboratory studies was also observed upon comparison of tissue accumulation factors across fish (Fig. 5(b)). For all fish tissue measurements, the laboratory estimations were generally lower than the in-field data. The medians of each tissue subgroup ranged from 10 to 494-fold higher for the in-field accumulation data when compared to laboratory data. The largest difference in medians belonged to estimations in bile. Bioaccumulation was observed to be relatively higher in liver, bile and brain tissue for both laboratory and in-field measurements. Greater partitioning of pharmaceuticals into these two tissues types might be expected since (a) the liver serves as the primary site of detoxification processes, and (b) there is selected transport of specific pharmaceuticals that have conserved molecular targets in the brain (e.g. antidepressants). It has been reported that antidepressants such as citalopram, sertraline and venlafaxine were detected at higher concentrations in the brains of fish when compared to other tissues (Grabicova et al., 2014; Lajeunesse et al., 2011).

Bile measurements had the largest median across all in-field subgroups. It may be expected that bile would display greater accumulation factors in comparison to other tissues, as xenobiotic detoxification often involves the transport of contaminants in bile into the bile canaliculi for either reabsorption or excretion. Many of the in-field bile accumulation estimates were derived from a single study focussing on antibiotic classes (Zhao et al., 2015). Biliary transport and excretion are an important elimination route for these types of pharmaceuticals (Karachalios and Charalabopoulos, 2002). It is interesting to consider that here bile serves as an elimination route containing excretory products and so demonstrates that fish can eliminate pharmaceuticals from the body. The relevance of bile measurement in occurrence and accumulation data is whether these pharmaceutical excretory products remain biologically active. In contrast to the in-field data, bile measurements represented the lowest median accumulation factors across all laboratory exposure tissue subgroups. Muscle tissue accumulation for the in-field data was the group that showed the second lowest median of 161.5 (lowest median was in plasma). In laboratory studies, muscle accumulation was also low with a median value of 2.88. The data indicates that distribution and accumulation of pharmaceuticals into muscle tissues is relatively low. This has been demonstrated in tissue specific accumulation studies (Zhao et al., 2015; Grabicova et al., 2014; Lajeunesse et al., 2011). However, pharmaceuticals have a relatively limited focus in the literature in this respect. This is of particular importance, as substances that have high accumulation in a specific tissue may still lead to deleterious effects and risk assessments based on whole-body accumulation factors might not describe this risk accurately as a result. Furthermore, biomonitoring studies that determine the internal pharmaceutical concentrations from muscle tissue may not give a reliable estimate of the extent of occurrence. As tissue distribution for pharmaceuticals in this compartment is likely to be low, potentially reflecting the blood flows and distribution depending on the compound, species and life stage.

An ANOVA analysis for fish tissue accumulation between laboratory and in-field data indicated that there was a statistical significance between the measured data (*p*-value = 2.394^−6^). A post-hoc analysis was performed using Tukey's test to identify which tissue subgroups (i.e. liver, gills, muscle etc.) led to the significant differences (See SI, Figure S3 and Table S3). Based on pairwise groupings, only two tissue-specific accumulation data groups were significantly different; the liver and bile. The significance may indicate that uptake in the field is more pronounced for these tissues when compared with laboratory-based testing. This disparity, among other factors mentioned above (such as environmental influences), could also arise due to the exposure in the field to large mixtures of pollutants rather than pharmaceuticals alone. Thus, exposure to complex mixtures may manifest through increased detoxification processes involving bile and the liver, leading to higher accumulation in these tissues. However, without high frequency sampling of surface waters, whether this significance is related to lower quality BAF estimates resulting from temporal fluctuations in contaminant surface water concentrations remains unclear.

## 10 Recommendations for future research

This review has summarised the occurrence of pharmaceuticals in biota to 2016, as well as emerging ways to potentially characterise the pharmaceutical component of the exposome more reliably. Several limitations still exist as identified in 2011, such as (a) limited data on tissue concentrations exist (b) ecotoxicity studies rarely report tissue concentrations and (c) limited breadth of target analytes (Beyer and Meador, 2011) indicating that progress within the field remains relatively slow. To further advance knowledge within environmental toxicology these gaps need to be urgently addressed and a number of other key issues have been identified since then. We propose the following set of recommendations which may begin to address existing and emergent gaps in knowledge, potentially towards improving pharmaceutical environmental risk assessments.

1) **Standardise analytical methods**, where possible, and adhere more strictly to method validation guidelines to ensure robust quantification. Harmonisation of the available guidelines for method validation that exist (Magnusson and. Örnemark, 2014; (ICH), 1994; Food and D, 2015; Committee, 1998; Thompson et al., 2002; Pharmacopoeia and U. and U., 2003) would enable movement away from method performance towards method validation. For example, pharmaceuticals in foodstuffs have been regulated for several years and conform to stringent validation criteria which could be adapted to environmental toxicology research. More specifically, guidelines for method validation using HRMS are also lacking. The importance of reliable and robust analytical methods within the field, is the foundation of which ecotoxicological studies should be based on.
2) **Measurement of internal concentrations** (including biotransformation products) in biota will enable more reliable risk assessment for pharmaceuticals in the environment than those based solely on concentrations in water (i.e. PEC/PNEC) and hence aid prioritisation of hazardous compounds. Internal concentrations are the initiating event for any potential pharmacological or toxicological effects and will be key to understanding risk. With respect to this, it is also advisable that effect-based studies should also quantify compound concentrations associated with the observed effects in biota. This will enable the establishment of the cause-effect relationship and threshold associated with the onset of the effect, and hence avoiding the vagaries of extrapolation of exposure concentrations to observed effects
3) **Tissue specific distribution** should also be determined, where possible, as single compartment measurements may not detect or artificially underestimate concentrations (via whole-body measurements). Preferential distribution of a compound into a specific tissue may cause localised effects, and so single compartment measurements may fail to characterise the potential risk to an organism. Advancements in mass spectrometric methods for imaging could be pursued to identify localisation of pharmaceuticals within an organism (Hsieh et al., 2007; Solon et al., 2010).
4) **Focus more on untargeted, hyphenated HRMS** analytical methods for screening purposes. The use of HRMS would also avoid biased pre-selection of contaminants (which may under-represent the true extent of environmental risk). Additionally, profiling in this way will also enable the elucidation of biotransformation pathways to inform toxicokinetic and effect-based assessments. The potential to simultaneously study complex mixtures, including metabolites and transformation products becomes possible towards better characterisation of the exposome and metabolome, for example. Of course, exposure at environmentally relevant concentrations are more likely to reflect the wild situations. Where technically possible these low concentrations would help avoid situations where concentration dependant uptake is an issue.
5) **Develop and validate new *in silico* approaches for mining** of so called ‘big data’ generated from untargeted methods should be urgently prioritised. With the previous recommendation of using HRMS, it is necessary to have tools that can expedite interpretation of these increasingly large and complex datasets. As ‘predictive ecotoxicology’ is becoming more well established, we must ensure that models are reliable and robust for these purposes.
6) **Improve reporting.** The reproducibility and replicability of studies could be improved by better standardised reporting of methods and data. For ionised compounds, the pH of the exposure media is critically important and, whilst often reported, it is not universally so. Similarly, concentration data from biomonitoring studies vary widely in presentation. For example, frequency is inconsistently given among reports. In addition, more of the exposure conditions should be reported (i.e. meta-data) to make the derived data of wider applicability for the future development of *in silico* models. It would also help avoid typographical errors if all publications used the same units of measurement when reporting concentrations in water.

Ultimately, reliable and robust analytical methods underpin understanding of the occurrence of pharmaceuticals in biota and the risks they may pose. Analytical capabilities are ever-increasing for broad-scope, targeted and untargeted biomonitoring which can be used in an interdisciplinary approach to characterise the pharmaceutical component of the exposome. Whilst the field of environmental toxicology has already seen a wealth of research in emerging contaminants, we remain unclear on the scale of pharmaceutical contamination and their potential combined effects on biota.

## Appendix A Supplementary data

SI Manuscript SI Manuscript
